# Characterization of adults concerning the use of a hypothetical mHealth application addressing stress-overeating: an online survey

**DOI:** 10.1186/s12889-024-18383-3

**Published:** 2024-04-04

**Authors:** Martin Lurz, Kathrin Gemesi, Sophie Laura Holzmann, Birgit Kretzschmar, Monika Wintergerst, Georg Groh, Markus Böhm, Kurt Gedrich, Hans Hauner, Helmut Krcmar, Christina Holzapfel

**Affiliations:** 1 Krcmar Lab (I17), Department of Computer Science, Technical University of Munich, Boltzmannstr. 3, 85748 Garching b. München, Germany; 2 Institute for Nutritional Medicine, School of Medicine & Health, Technical University of Munich, Munich, Germany; 3 Research Group Public Health Nutrition, ZIEL– Institute for Food & Health, Technical University of Munich, Freising, Germany; 4 Research Group Social Computing, Department of Computer Science, Technical University of Munich, Garching, Germany; 5 Department of Informatics, University of Applied Sciences Landshut, Landshut, Germany; 6 Else Kröner Fresenius Center for Nutritional Medicine, ZIEL - Institute for Food & Health, Technical University of Munich, Freising, Germany; 7 Department of Nutritional, Food and Consumer Sciences, Fulda University of Applied Sciences, Fulda, Germany

**Keywords:** Nutrition, Digital tool, Behavioral intention, Obesity

## Abstract

**Background:**

About 40% of people respond to stress by consuming more unhealthy foods. This behavior is associated with increased energy intake and the risk of obesity. As mobile health (mHealth) applications (apps) have been shown to be an easy-to-use intervention tool, the characterization of potential app users is necessary to develop target group-specific apps and to increase adherence rates.

**Methods:**

This cross-sectional online survey was conducted in the spring of 2021 in Germany. Sociodemographic data and data on personality (*Big Five Inventory*, BFI-10), stress-eating (*Salzburg Stress Eating Scale*, SSES), and technology behavior (*Personal Innovativeness in the Domain of Information Technology*, PIIT; *Technology Acceptance Model 3*, TAM 3) were collected.

**Results:**

The analysis included 1228 participants (80.6% female, mean age: 31.4 ± 12.8 years, mean body mass index (BMI): 23.4 ± 4.3 kg/m^2^). Based on the TAM score, 33.3% (409/1228) of the participants had a high intention to use a hypothetical mHealth app to avoid stress-overeating. These persons are characterized by a higher BMI (24.02 ± 4.47 kg/m^2^, *p* < 0.001), by being stress-overeaters (217/409, 53.1%), by the personality trait “neuroticism” (*p* < 0.001), by having specific eating reasons (all *p* < 0.01), and by showing a higher willingness to adopt new technologies (*p* < 0.001).

**Conclusion:**

This study suggests that individuals who are prone to stress-overeating are highly interested in adopting an mHealth app as support. Participants with a high intention to use an mHealth app seem to have a general affinity towards new technology (PIIT) and appear to be more insecure with conflicting motives regarding their diet.

**Trial registration:**

This survey was registered in the German Clinical Trials Register (Registration number: DRKS00023984).

## Introduction

Stress is the temporary inability of an individual’s body to find a specific and certain response to a perceived load that exceeds their coping skills [1]. The number of people suffering from stress reached new highs in 2020 and again in 2021, which may have been aggravated by the COVID-19 pandemic [2]. According to the American Psychological Association, over 80% of the American adult population reported prolonged stress in 2020 [3]. Data from 2021 showed that 64% of the population in Germany feels stressed at least sometimes [4].

Stress also affects eating behavior [5–8]. About 80% of the population change their eating behavior when stressed, with one half tending to eat less and the other half showing hyperphagic behavior. Hyperphagia hereby refers to the excessive consumption of food (= stress-overeating) associated with an increased energy intake [9]. However, not only do quantities increase, but also the type of food changes, as people under stress tend to eat food high in sugar, fat, and energy, such as chocolate or fast food (= comfort foods) [10, 11]. In the Adult Study of the Leipzig Research Centre for Civilization Diseases (LIFE) a relationship between stress and food addiction has been shown [12]. Moreover, some results indicate that people store more fat under stress than when relaxed [13]. For this reason, stress-overeating is often considered one of the reasons for the development of obesity [14], which is a major driver for chronic diseases [15] and non-communicable diseases such as cardiovascular diseases [16], certain types of cancer [17], and diabetes mellitus [16]. While the increased food intake is suspected to be caused by changes in hormone levels due to stress [10, 18], it has to be noted, that stress-overeating can also be a symptom of an eating disorder such as Binge Eating [19]. Furthermore, it is well known, that stress is associated with Binge Eating [20].

With the growing popularity of smartphones, mobile health (mHealth) applications (apps) have shown to be both a low-cost and a generally well-accepted intervention tool [21, 22]. While from a clinical point of view, the treatment of complex diseases such as eating disorders requires more than an mHealth app, self-monitoring apps showed a change in user behavior towards healthier food choices [23]. However, currently available nutrition apps primarily focus on tracking food intake and giving feedback on a calorie-based level while not taking users’ circumstances (e.g., stress) into account [24]. Therefore, digital support in stressful situations, which could lead to stress-overeating, is currently unavailable. Furthermore, current mHealth apps often suffer from low adherence rates [25, 26]. One reason might be that such applications are not tailored to the needs of specific target groups [27], such as stress-overeaters. Additionally, only limited information is available for this target group [28].

This secondary analysis of an online survey [11, 29] aimed to characterize people interested in using a mHealth app for managing stress-overeating in a hypothesis-free approach. Characteristics are based on sociodemographics, anthropometrics, stress-eating behavior, eating reasons, and personality traits.

## Methods

### Survey

People throughout Germany were invited to participate in the open online survey between January and April 2021. According to the Covid Stringency Index, which was between 75 and 82.5 when the survey was conducted [30], the government policies in Germany were quite strict at that time (100 = strictest). Nevertheless, a strong Covid-related bias is not assumed regarding this data since the survey was conducted online. Furthermore, current stress levels were not asked and questions were focused on the acceptance of a hypothetical app. Participants were recruited digitally using university internal and external channels (e.g., newsletters, social media, and email). Participants were invited to the online survey, including a link to the survey platform SoSci Survey (V3.1.06). They had to confirm the data privacy statement and give informed consent before participating in the survey. Details on the survey methodology have been published previously [11, 28].

### Questionnaire

The questionnaire was developed with a team including nutritionists, public health experts as well as computer scientists. The final set included 38 questions (closed, open, single, or multiple choice) about nutrition (1 question), perception and coping with stress (4 questions), stress-induced eating (17 questions), technology usage behavior (4 questions), app acceptance (3 questions), and personality (1 question). Furthermore, demographic and anthropometric data (8 questions) were collected. Each question was mandatory to be answered before being able to continue. However, participants could end the survey at any point.

The current analysis focuses on the data about the acceptance of a hypothetical mHealth app that deals with stress-overeating. Participants were given a short description of a hypothetical app that would be able to recognize stress and predict stressful situations by measuring physiological parameters like pulse and tracking other data (e.g., location, number of diary entries, sleep quality). In addition, this app could support users in maintaining healthy nutrition behavior by suggesting healthier alternatives to comfort foods [11].

### Stress-eating behavior

For the characterization of stress-eaters, the validated *Salzburg Stress Eating Scale* (SSES) with proven one-factorial, internal consistency and convergent validity was used [31]. On a 5-level Likert scale, participants answered how they respond to stressful events. Participants were categorized as “eats more when stressed” (stress-overeaters, score > 3), “eats less when stressed” (stress-undereaters, score < 3), and “eats the same amount as usual” (non-stress eaters, score = 3) [31].

### Personality

The participants’ personality traits were assessed by using the validated *Big Five Inventory* (BFI-10) questionnaire with proven objectivity, reliability, and validity (content, factorial, and construct) [32]. The five dimensions “Agreeableness,” “Conscientiousness,” “Extraversion,” “Neuroticism,” and “Openness” are displayed each by two items. A mean score was calculated per personality trait. Higher mean scores indicate a greater contribution of the respective trait to the participants’ personalities.

### Eating reasons

To assess why participants eat what they eat, a subset of 15 items of the validated *The Eating Motivation Survey* (TEMS) with proven reliability and validity (construct, convergent, and discriminant) [33] was included. Each item represents a different reason to eat. A 7-level Likert scale (1 = never to 7 = always) was provided per item. Higher mean scores indicate a greater importance of the respective eating reason. Due to missing data, subscales were not calculated.

### Personal innovativeness

The participants’ attitude towards new technologies (= hypothetical mHealth app), was examined with the validated *Personal Innovativeness in the Domain of Information Technology* (PIIT) questionnaire with proven reliability [34]. Four items were presented to the participants, each with a 5-level Likert scale. A mean score was calculated over all items. Higher scores indicate a more positive attitude towards the hypothetical mHealth app.

### Acceptance of the hypothetical app

To assess the acceptance of the described hypothetical mHealth app, the validated *Technology Acceptance Model 3* (TAM 3) with proven reliability and validity (convergent and discriminant) [35] was chosen. The two dimensions, “Perceived Usefulness” (PU, represented by 4 items) and “Behavioral Intention” (BI, represented by 3 items), were used. Per item, participants answered on a 5-level Likert scale. Mean scores were calculated for PU and BI separately and for both dimensions together. A higher mean score indicates a greater perceived usefulness or behavioral intention to use or, in general, a greater acceptance of the hypothetical app. For a detailed characterization of potential users, participants were categorized in terms of their intention to use the hypothetical app (BI). Therefore, the sample was divided into three equal groups by creating tertiles (low BI: *n* = 410; medium BI: *n* = 409, high BI: *n* = 409).

### Statistical analysis

As a first step, integrity and plausibility checks were performed. For this hypothesis-free analysis, completers based on the variables of interest were included. In total, 1480 persons started to answer the survey and provided an answer at least for one question out of the 38 questions. Data from 1228 participants from whom also sociodemographic data are available, were included in the analysis. Descriptive data analyses (frequencies, percentages, mean, and standard deviation (SD)) were performed. Differences in mean were estimated by performing the Kruskal-Wallis rank sum test and Wilcoxon rank sum test. Pearson’s Chi-squared test was used to examine differences in the distribution of categorical variables. P-values calculated for the variables “Personality” and “Eating reasons” were adjusted for multiple testing according to Bonferroni-Holm [36]. P-values < 0.05 were considered indicating statistically significant results. All analyses were performed using R version V1.4.1717 (R Core Team, 2020, http://www.r-project.org).

## Results

### General population characteristics

As shown in Table 1, data from 1228 participants were included in the analysis. The majority of the participants (990/1228, 80.6%) were female. The age ranged from 18 to 82 years, with the average participant being 31.4 ± 12.8 years old and having a BMI of 23.4 ± 4.3 kg/m^2^. Based on the results of the SSES, 41.8% (513/1228) of the participants were stress-overeaters, 45.9% (564/1228) were stress-undereaters, and 12.3% (151/1228) were non-stresseaters.

**Table 1:** General population characteristics

Characteristic	Number (%) of participants	Mean ± SD
**Gender**		
Male	238 (19.4%)	-
Female	990 (80.6%)	-
**Age** (years)	-	31.4 ± 12.8
**BMI** (kg/m^2^)	-	23.4 ± 4.3
**Occupation**		
Student	658 (53.3%)	-
Employee	431 (34.9%)	-
Other	145 (11.8%)	-
**Stress-eating** (*SSES*)		
Stress-overeaters (SSES > 3)	513 (41.8%)	3.69 ± 0.45
Stress-undereaters (SSES < 3)	564 (45.9%)	2.41 ± 0.42
Non-stresseaters (SSES = 3)	151 (12.3%)	3.00

*Note N* = 1228. BMI = body mass index; SD = standard deviation; SSES = Salzburg Stress Eating Scale

### Characterization of SSES groups

**Fig. 1:**
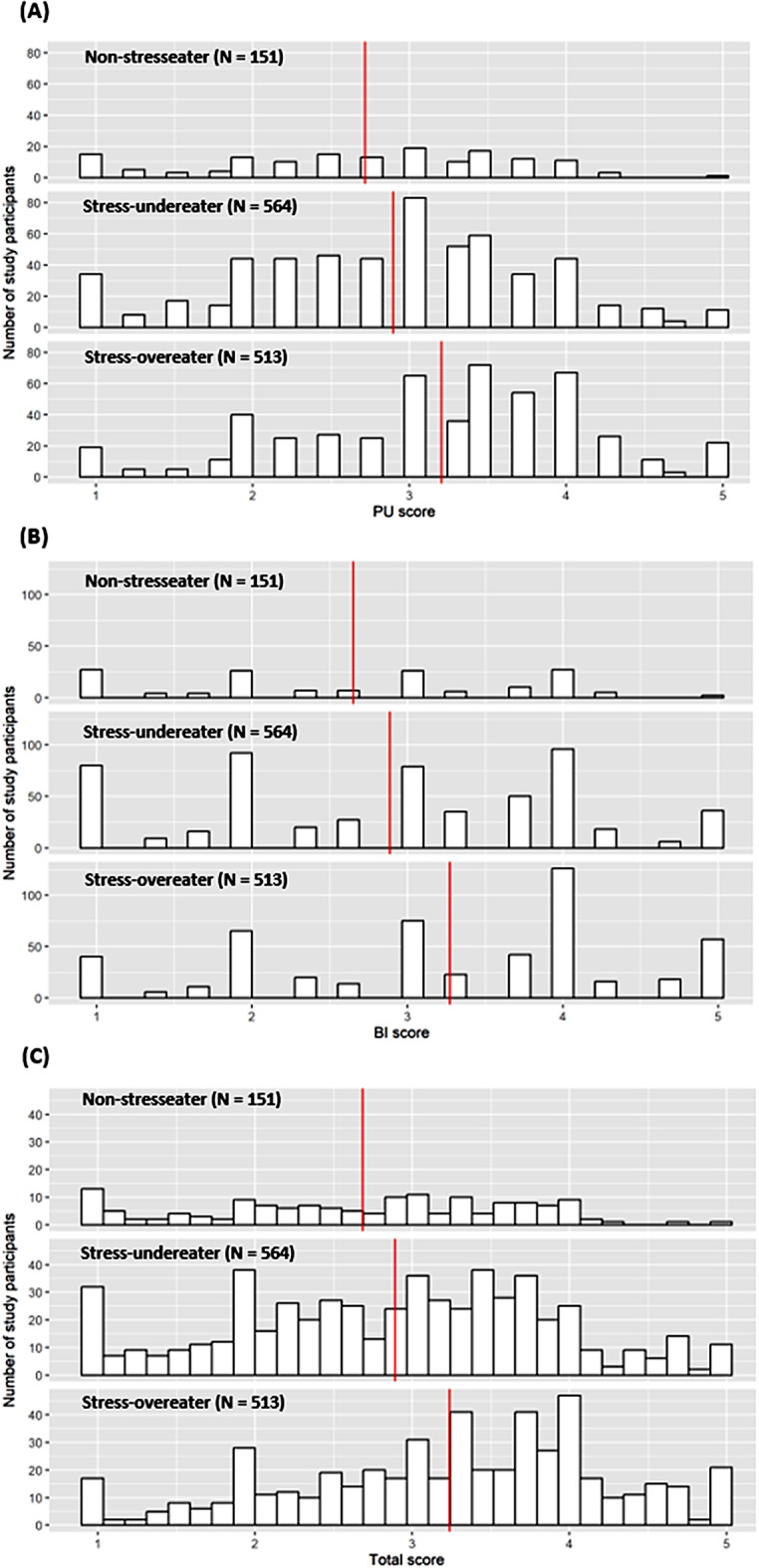
Characterization of SSES groups according to **(A)** PU, **(B)** BI, and **(C)** total score. *Note* This figure shows number of study participants on the y-axis and scoring frequency (PU, BI, and total score summarizing PU and BI) on the x-axis with the mean score (red line) per SSES group. BI = Behavioral Intention; PU = Perceived Usefulness; SSES = Salzburg Stress Eating Scale

To gain insight into the hypothetical app acceptance based on stress-eating behavior, the study population was categorized by their SSES scores. As shown in Fig. 1, ratings for PU (Fig. 1A), BI (Fig. 1B), as well as the resulting total score (Fig. 1C) differed between the three SSES groups. On average stress-overeaters rated all three scores significantly higher (PU score = 3.20 ± 0.93; BI score = 3.27 ± 1.16; total score = 3.24 ± 0.98) compared to stress-undereaters (PU score = 2.89 ± 0.93; BI score = 2.89 ± 1.18; total score = 2.89 ± 1.00) or non-stresseaters (PU score = 2.72 ± 0.93; BI score = 2.65 ± 1.12; total score = 2.68 ± 0.97), with all p-values < 0.001.

### Characterization of BI groups

Table 2 shows the characterization of participants with low, medium, and high BI as a proxy for technical acceptance. The distribution of gender between the three BI groups was statistically significantly different (*p* = 0.002). There was no significant difference in age (*p* = 0.26).

The mean BMI was significantly different between the three BI groups (*p* < 0.001): BMI of the low BI group = 22.63 ± 3.76 kg/m^2^, BMI of the medium BI group = 23.50 ± 4.54 kg/m^2^, BMI of the high BI group = 24.02 ± 4.47 kg/m^2^. The portion of participants with BMI < 18.5 kg/m^2^ was the greatest in the low BI group (38/410, 9.3% vs. 16/409, 3.9% and 11/409, 2.7%). The portion of participants with the highest BMI category (≥ 30.0 kg/m^2^) was the greatest in the high BI group (18/410, 4.5% in low BI group vs. 36/409, 8.8% in medium BI group and 41/409, 10.0% in high BI group) (Table 2).

The SSES score significantly differed between the low BI and high BI groups and the three BI groups (*p* < 0.001). Furthermore, the distribution of stress-eaters was significantly different between the three BI groups (*p* < 0.001). The low BI group consisted of 32.9% (135/410), the medium BI group of 39.4% (161/409), and the high BI group of 53.1% stress-overeaters (217/409). Stress-undereaters and non-stresseaters were distributed in a reversed order (Table 2).

For personality, the degree of “neuroticism” was found to be significantly different between the BI groups as well as for comparing the low BI group to the high BI group (*p* < 0.001) with the lowest degree in the low BI group and the highest degree in the high BI group (3.09 ± 0.93 vs. 3.48 ± 0.90, *p* < 0.001) (Table 2).

Ratings of five of the 15 eating reasons showed statistically significant differences between the BI groups (all *p* < 0.05). For all five eating reasons, the high BI group scored highest and the low BI group scored lowest (Table 2).

Scores of personal innovativeness were significantly different between the BI groups (*p* < 0.001), with being lowest for the low BI group and highest for the high BI group (2.50 ± 0.92 vs. 3.13 ± 0.95, *p* < 0.001) (Table 2).

**Table 2:** *Characterization of participants based on low, medium, or high BI score*

Characteristics	Low BI (*n* = 410)	Medium BI (*n* = 409)	High BI (*n* = 409)	Group differences
	mean ± SD or n (%)	mean ± SD or n (%)	mean ± SD or n (%)	p-value (p-value^c^ low BI vs. high BI)
**Gender** ^a^				
Male	101 (24.6)	75 (18.3)	62 (15.2)	0.002
Female	309 (75.4)	334 (81.7)	347 (84.8)	
**Age** (years)^b^	31.26 ± 12.86	31.78 ± 12.92	31.38 ± 12.68	0.26
18–29	271 (66.1)	254 (62.1)	249 (60.9)	
30–49	77 (18.8)	88 (21.5)	106 (25.9)	
50–64	55 (13.4)	62 (15.2)	49 (12.0)	
≥ 65	7 (1.7)	5 (1.2)	5 (1.2)	
**BMI** (kg/m^2^)^b^	22.63 ± 3.76	23.50 ± 4.54	24.02 ± 4.47	< 0.001 (< 0.001)
< 18.5	38 (9.3)	16 (3.9)	11 (2.7)	
18.5–24.9	283 (69.0)	290 (70.9)	262 (64.1)	
25.0–29.9	71 (17.3)	67 (16.4)	95 (23.2)	
≥ 30.0	18 (4.4)	36 (8.8)	41 (10.0)	
**Stress-eating behavior** (SSES)^b^	2.90 ± 0.62	2.97 ± 0.70	3.17 ± 0.82	< 0.001 (< 0.001)
Stress-overeaters	135 (32.9)	161 (39.4)	217 (53.1)	< 0.001
Stress-undereaters	209 (51.0)	197 (48.2)	158 (38.6)	
Non-stresseaters	66 (16.1)	51 (12.5)	34 (8.3)	
**Personality** (BFI-10)^b^				
Agreeableness	3.18 ± 0.76	3.24 ± 0.75	3.26 ± 0.79	0.56
Extraversion	3.20 ± 1.03	3.24 ± 0.75	3.26 ± 0.79	0.63
Conscientiousness	3.68 ± 0.81	3.58 ± 0.78	3.59 ± 0.83	0.46
Neuroticism	3.09 ± 0.93	3.26 ± 0.92	3.48 ± 0.90	< 0.001 (< 0.001)
Openness	3.65 ± 0.94	3.50 ± 0.94	3.60 ± 0.96	0.25
**Eating reasons** (I eat what I eat because…)^b^				
I like it.	6.26 ± 0.90	6.21 ± 0.93	6.24 ± 0.84	1
I usually eat it.	5.02 ± 1.33	5.00 ± 1.28	5.19 ± 1.25	0.30
I’m hungry.	5.55 ± 1.24	5.45 ± 1.13	5.48 ± 1.20	0.65
it is healthy.	5.22 ± 1.22	5.16 ± 1.22	5.23 ± 1.16	1
it is the most convenient.	4.13 ± 1.54	4.40 ± 1.44	4.56 ± 1.52	0.002 (0.001)
to indulge myself.	4.73 ± 1.45	4.93 ± 1.29	5.07 ± 1.27	0.052
out of traditions.	3.55 ± 1.71	3.79 ± 1.66	3.76 ± 1.76	0.65
it is natural.	4.33 ± 1.79	4.37 ± 1.77	4.27 ± 1.80	1
it is social.	4.11 ± 1.60	4.41 ± 1.60	4.25 ± 1.68	0.18
it is inexpensive.	3.26 ± 1.56	3.42 ± 1.51	3.45 ± 1.58	0.65
it spontaneously appeals to me.	3.27 ± 1.55	3.64 ± 1.55	3.86 ± 1.60	< 0.001 (< 0.001)
I watch my weight.	3.71 ± 1.82	3.97 ± 1.67	4.29 ± 1.68	< 0.001 (< 0.001)
I am frustrated.	2.78 ± 1.72	3.26 ± 1.78	3.86 ± 1.88	< 0.001 (< 0.001)
I am supposed to eat it.	1.91 ± 1.30	2.18 ± 1.37	2.28 ± 1.49	0.003 (0.002)
others like it.	1.71 ± 1.04	1.90 ± 1.22	2.01 ± 1.31	0.052
**Personal innovativeness** ^b^	2.50 ± 0.92	2.82 ± 0.85	3.13 ± 0.95	< 0.001 (< 0.001)

*Note* BFI = Big Five Inventory; BI = Behavioral Intention; BMI = body mass index; SD = standard deviation; SSES = Salzburg Stress Eating Scale

^a^Categorical variable, Pearson’s Chi squared test used for comparison

^b^Not normally distributed, Kruskal-Wallis rank sum test used for comparison

^c^Wilcoxon rank sum test used for pairwise comparison

## Discussion

In this study, the target group of a hypothetical mHealth app addressing stress-overeating was characterized. Three groups significantly differing in several characteristics were hereby determined on their ratings for the intention to use such an app. As all three BI groups include participants with a high prevalence of stress-induced eating, a specific analysis of the different groups is necessary.

The evaluation of the TAM dimensions among the three different stress-eating groups showed that stress-overeaters rated PU and BI the highest compared to the other two stress-eating groups. This indicates that most participants prone to stress-overeating would find it useful to have a mHealth app supporting a healthier eating behavior in stressful situations. The value of PU in this survey is comparable to the rating of a digital diary-based nutrition app, which has been used in a study [37]. It has to be mentioned that in this survey the rating refers to a hypothetical app described in the methods part and, therefore, participants have not used the mHealth app dealing with stress-overeating. Since it might be difficult for users to grasp the usefulness of something they have never tried before, PU ratings might increase if people could actually use the app in the real world. As BI has been found to be directly influenced by PU in the acceptance model [35], the high value in this group is comprehensible.

The survey data showed a significant gender difference. The greater interest of women in the supposed mHealth app of this research confirms prior results of publications in this area. Former studies have shown that females tend to be more concerned with their diet and, consequently more determined to self-regulate their eating behavior than males [38].

Despite the highest average BMI of users in the high BI group, detailed analysis showed that persons in different BMI groups showed interest in the theoretical app. Therefore, we must emphasize that recommendations based purely on dietary energy, as it is common in many nutrition apps [24], should not be the focus in managing stress-overeating.

Regarding the different personality traits, values for neuroticism– mainly associated with anxiousness, embarrassment, and insecurity [39]– were significantly different between the three BI groups, with the highest score among the participants of the high BI group. Previous research has shown that unhealthy food choices and overeating is also associated with neuroticism [40], explaining the high percentage of stress-overeaters in the high BI group. These results are supported by other studies which showed a positive correlation between neuroticism and the usage of behavior change apps in the area of physical activity [41] and health promotion services [42].

Looking at eating reasons, e.g., convenience (“I eat what I eat because it is the most convenient. “) was prominently high for the high BI group. This eating reason matches the definition of comfort foods (such as chocolate, ice cream, or coffee) but also typical convenience foods (such as fries and similar fast food) [11].

Eating patterns are provoked by stress [43] and negative emotions can lead to emotional eating or rather the consumption of energy-dense comfort food [11, 44]. In the long term, frequent stress-overeating of unhealthy food products could increase body weight. Once (unhealthy) eating habits are established, they are hard to break [45]. At this point, the described mHealth app could recommend healthier food alternatives like fruits, vegetables, and nuts in moments of stress [11]. These recommendations could help to establish a healthier stress-eating behavior.

Finally, the high ratings for personal innovativeness [34] in the high BI group are in line with prior research, which showed that personal innovativeness influences BI [46]. The significant difference between the groups indicates that non-usage of a mHealth app addressing stress-overeating might not be due to general disinterest in nutrition support but that people with lower intention to use such an app would maybe require more encouragement and technical support to have the confidence and trust to implement such an app in their daily life. Thus, we suggest providing onboarding tutorials to lower entry barriers [47].

Overall, the expected characteristics of our target group (= high BI group) for a potential app dealing with stress-overeating could be confirmed. Higher scoring in “neuroticism” and the observed eating reasons go well with the findings that this group tends to strongly represent stress-overeaters and have a higher BMI. Consequently, a mHealth app for stress-overeating avoidance should not only target the two factors stress-eating behavior and BMI, but also consider the users’ personality and eating reasons.

The present findings are based on the intention to use an app, which can be seen as the first step towards successful use. Nevertheless, adherence is most important. For continuous use, risk perception and performance expectation [48] as well as user experience and intent [49], next to influencing factors already presented in this paper such as age and perceived usefulness [50] are key. In future app developments such values should be considered.

Our findings are not without limitations. As data were collected through a questionnaire, response bias might apply. Furthermore, the study population with a large group of young females is not representative of the overall German population. However, our sample showed a similar distribution of stress-overeaters as found in previous research [51]. The analysis regarding app usage was conducted on an intentional level without obtaining real usage data of a mobile application. The groups based on intention to use were empirically driven based on the available data. For this reason, average values in the groups may be distorted. This might be the case especially in the group with the lowest intention, as participation in the survey already requires a certain level of interest. Furthermore, the analysis is focused on fully completed questionnaires (based on the values needed). To characterize eating motivation single items of the TEMS questionnaire were analyzed. Additional assessment of eating behaviour is missing. Restrictions may also apply to other items as all data are self-reported. Finally, data collection took place during COVID-19-pandemic, which may have an impact on the data on perceived stress.

Nevertheless, our research contributes to theory and practice in multiple areas. It extends the research on characteristics of people prone to stress-eating [28] by providing a new perspective through insights into the acceptance of mHealth apps addressing stress-overeating.

## Conclusion

This survey showed the general interest in an mHealth app addressing stress-overeating of the main target group, which consists mainly of people prone to stress-overeating. Our characterization draws the average image of people on a more insecure side with contradictory motives regarding their diet, vacillating between comfort and control, and having a general affinity for and the willingness to adopt new technologies. This suggests that potential users may have the intrinsic motivation for positive eating behavior change but need support to implement it. In this context, a mHealth app focusing on stress-overeating should consider peoples’ demographics, eating reasons, and personality to pursue a sustainable strategy for adopting a healthier eating behavior in stressful situations.

## Data Availability

The datasets used and/or analyzed during the current study are available from the corresponding author on reasonable request.

## References

[1] Selye H (1956). The stress of life.

[2] Gallup. Gallup Global emotions 2022. Gallup, Inc.; 2022.

[3] Canady VA (2021). APA survey: majority of americans reporting prolonged stress. Mental Health Wkly.

[4] Techniker Krankenkasse (2021). Entspann dich, Deutschland! TK-Stressstudie 2021.

[5] American Psychology Association. Stress in AmericaTM: our health at risk. 2012.

[6] Dallman MF (2010). Stress-induced obesity and the emotional nervous system. Trends Endocrinol Metabolism.

[7] Stone AA, Brownell KD (1994). The stress-eating paradox: multiple daily measurements in adult males and females. Psychol Health.

[8] Torres SJ, Nowson CA (2007). Relationship between stress, eating behavior, and obesity. Nutrition.

[9] Halford JCG, Stolerman IP (2010). Hyperphagia. Encyclopedia of psychopharmacology.

[10] Adam TC, Epel ES (2007). Stress, eating and the reward system. Physiol Behav.

[11] Gemesi K, Holzmann SL, Kaiser B, Wintergerst M, Lurz M, Groh G (2022). Stress eating: an online survey of eating behaviours, comfort foods, and healthy food substitutes in German adults. BMC Public Health.

[12] Hussenoeder FS, Conrad I, Löbner M, Engel C, Reyes N, Yahiaoui-Doktor M (2023). The different areas of chronic stress and food addiction: results from the LIFE-Adult-study. Stress Health.

[13] Björntorp P. Do stress reactions cause abdominal obesity and comorbidities? Obes Rev. 2001;2(2):73–86. Epub 2002/07/18. 10.1046/j.1467-789x.2001.00027.x. PubMed PMID: 12119665. 10.1046/j.1467-789x.2001.00027.x 12119665

[14] Block JP, He Y, Zaslavsky AM, Ding L, Ayanian JZ (2009). Psychosocial stress and change in weight among US adults. Am J Epidemiol.

[15] Health Effects of Overweight and Obesity in 195 Countries over 25 Years. New England Journal of Medicine. 2017;377(1):13–27. 10.1056/NEJMoa1614362. PubMed PMID: 28604169. 10.1056/NEJMoa1614362 PMC5477817 28604169

[16] Singh GM, Danaei G, Farzadfar F, Stevens GA, Woodward M, Wormser D (2013). The age-specific quantitative effects of metabolic risk factors on cardiovascular diseases and diabetes: a pooled analysis. PLoS ONE.

[17] Lauby-Secretan B, Scoccianti C, Loomis D, Grosse Y, Bianchini F, Straif K (2016). Body fatness and cancer—viewpoint of the IARC Working Group. N Engl J Med.

[18] Peters A, Pellerin L, Dallman MF, Oltmanns KM, Schweiger U, Born J (2007). Causes of obesity: looking beyond the hypothalamus. Prog Neurobiol.

[19] Laessle RG, Schulz S (2009). Stress-induced laboratory eating behavior in obese women with binge eating disorder. Int J Eat Disord.

[20] Naish KR, Laliberte M, MacKillop J, Balodis IM (2019). Systematic review of the effects of acute stress in binge eating disorder. Eur J Neurosci.

[21] Modrzejewska J, Modrzejewska A, Czepczor-Bernat K, Matusik P (2022). The role of body mass index, healthy eating-related apps and educational activities on eating motives and behaviours among women during the COVID-19 pandemic: a cross sectional study. PLoS ONE.

[22] Payne HE, Lister C, West JH, Bernhardt JM (2015). Behavioral functionality of mobile apps in health interventions: a systematic review of the literature. JMIR mHealth uHealth.

[23] Dunn CG, Turner-McGrievy GM, Wilcox S, Hutto B (2019). Dietary self-monitoring through calorie tracking but not through a digital photography app is associated with significant weight loss: the 2SMART pilot study—A 6-month randomized trial. J Acad Nutr Dietetics.

[24] Leipold N, Lurz M, Wintergerst M, Groh G. Goal-Setting Characteristics of Nutrition-Related mHealth Systems: A Morphological Analysis. 2022.

[25] Perez S. Flurry Examines App Loyalty: News & Communication Apps Top Charts, Personalization Apps See High Churn: TechCrunch; 2012 [October 20, 2021]. Available from: https://techcrunch.com/2012/10/22/flurry-examines-app-loyalty-news-communication-apps-top-charts-personalization-apps-see-high-churn/.

[26] Thompson FE, Subar AF. Dietary assessment methodology. In: Coulston AM, Boushey CJ, Ferruzzi MG, Delahanty LM, editors. Nutrition in the Prevention and Treatment of Disease. Academic; 2017. p. 1072.

[27] König LM, Attig C, Franke T, Renner B. Barriers to and Facilitators for Using Nutrition Apps: Systematic Review and Conceptual Framework. JMIR Mhealth Uhealth. 2021;9(6). Epub 2021/07/14. 10.2196/20037. PubMed PMID: 34254938; PubMed Central PMCID: PMCPMC8409150. 10.2196/20037 PMC8409150 34254938

[28] Kaiser B, Gemesi K, Holzmann SL, Wintergerst M, Lurz M, Hauner H (2022). Stress-induced hyperphagia: empirical characterization of stress-overeaters. BMC Public Health.

[29] Kaiser B, Holzmann S, Hauner H, Holzapfel C, Kurt G (2020). Nutrition and stress: overview of selected stress indicators and smart measurment techniques. Ernahrungs-Umschau.

[30] Hale T, Angrist N, Goldszmidt R, Kira B, Petherick A, Phillips T (2021). A global panel database of pandemic policies (Oxford COVID-19 Government Response Tracker). Nat Hum Behav.

[31] Meule A, Reichenberger J, Blechert J (2018). Development and preliminary validation of the Salzburg Emotional Eating Scale. Front Psychol.

[32] Rammstedt B, Kemper CJ, Klein MC, Beierlein C, Kovaleva A. A short scale for assessing the big five dimensions of personality: 10 item big five inventory (BFI-10). 2013;7(2). 10.12758/mda.2013.013.

[33] Renner B, Sproesser G, Strohbach S, Schupp HT (2012). Why we eat what we eat. The eating motivation survey (TEMS). Appetite.

[34] Agarwal R, Prasad J (1998). A conceptual and operational definition of personal innovativeness in the domain of information technology. Inform Syst Res.

[35] Venkatesh V, Bala H (2008). Technology acceptance model 3 and a research agenda on interventions. Decis Sci.

[36] Hemmerich W, StatistikGuru. Rechner zur Adjustierung des α-Niveaus 2016 [March 03, 2022]. Available from: https://statistikguru.de/rechner/adjustierung-des-alphaniveaus.html.

[37] Hauptmann H, Leipold N, Madenach M, Wintergerst M, Lurz M, Groh G (2022). Effects and challenges of using a nutrition assistance system: results of a long-term mixed-method study. User Model User-Adapt Interact.

[38] Hamilton T, Hoffman J, Arsiwalla D, Volpe R, Schmidt E, Gropper S. Gender comparisons of young adults’ eating behavior regulation: Re-examination of the Regulation of Eating Behavior Scale (REBS). Appetite. 2018;126. 10.1016/j.appet.2018.03.014. Epub 2018/04/01.:80– 9. 10.1016/j.appet.2018.03.014 29604318

[39] Digman JM (1990). Personality structure: emergence of the five-factor model. Ann Rev Psychol.

[40] Keller C, Siegrist M (2015). Does personality influence eating styles and food choices? Direct and indirect effects. Appetite.

[41] Belmon LS, Middelweerd A, te Velde SJ, Brug J (2015). Dutch young adults ratings of Behavior Change techniques Applied in Mobile phone apps to promote physical activity: a cross-sectional survey. JMIR mHealth uHealth.

[42] Bregenzer A, Wagner-Hartl V, Jiménez P (2017). Who uses apps in health promotion? A target group analysis of leaders. Health Inf J.

[43] Pool E, Delplanque S, Coppin G, Sander D (2015). Is comfort food really comforting? Mechanisms underlying stress-induced eating. Food Res Int.

[44] Dubé L, LeBel JL, Lu J (2005). Affect asymmetry and comfort food consumption. Physiol Behav.

[45] Heymsfield SB, Harp JB, Reitman ML, Beetsch JW, Schoeller DA, Erondu N (2007). Why do obese patients not lose more weight when treated with low-calorie diets? A mechanistic perspective. Am J Clin Nutr.

[46] Koivisto K, Makkonen M, Frank L, Riekkinen J, editors. Extending the technology acceptance model with personal innovativeness and technology readiness: a comparison of three models. Bled eConference; 2016: Moderna organizacija.

[47] Joyce G, Lilley M, Barker T, Jefferies A, editors. Mobile Application tutorials: perception of usefulness from an HCI Expert Perspective. Human-Computer Interaction Interaction Platforms and Techniques; 2016.

[48] Wei J, Vinnikova A, Lu L, Xu J (2021). Understanding and Predicting the Adoption of Fitness Mobile apps: evidence from China. Health Commun.

[49] Vaghefi I, Tulu B (2019). The continued use of mobile health apps: insights from a longitudinal study. JMIR mHealth uHealth.

[50] Wang T, Wang W, Liang J, Nuo M, Wen Q, Wei W (2022). Identifying major impact factors affecting the continuance intention of mHealth: a systematic review and multi-subgroup meta-analysis. Npj Digit Med.

[51] Oliver G, Wardle J (1999). Perceived effects of stress on food choice. Physiol Behav.

